# Cutaneous geotrichosis due to *Geotrichum candidum* in a burn patient

**DOI:** 10.1099/acmi.0.000001

**Published:** 2019-03-20

**Authors:** Sarah Keene, Manbeer S. Sarao, Philip J. McDonald, Jennifer Veltman

**Affiliations:** 1 Department of Internal Medicine, Wayne State University, Detroit, MI, USA; 2 Armed Forces Medical College, Pune, Maharashtra, India; 3 Division of Infectious Diseases, Department of Internal Medicine, Wayne State University, Detroit, MI, USA

**Keywords:** geotrichosis, burn, immunocompromise, invasive fungal infection, antifungal therapy

## Abstract

*Geotrichum candidum* is a saprophytic yeast known to colonize the human skin, respiratory tract and gastrointestinal tract. It can cause local or disseminated disease (geotrichosis), mainly in the immunocompromised host. Trauma, indwelling catheter use, prolonged broad-spectrum antibiotic treatment and critical illness have also been implicated as risk factors. Here we report the first case, to our knowledge, of cutaneous *G. candidum* infection in a burn patient. The isolate had a high amphotericin B minimum inhibitory concentration (MIC) and the patient experienced concomitant *Candida* *orthopsilosis* fungaemia, and so was treated with a combination of voriconazole and micafungin. This case highlights the importance of source control, rapid identification of *G. candidum* infection and MIC determination to guide antifungal therapy, which typically consists of amphotericin B with or without flucytosine or voriconazole alone. Clinicians should be aware of geotrichosis as a clinical entity in burn patients as well as in the immunocompromised. Antifungal resistance and breakthrough disease are an ongoing concern due to the increasing number of immunocompromised at-risk patients and the use of routine mould prophylaxis.

## Introduction

*Geotrichum candidum* (teleomorph: *Galactomyces candidus*) is a ubiquitous, dimorphic, ascomycetous yeast of the *Saccharomycetes* class [1]. This saprophytic organism is known to cause post-harvest rot in a number of fruit and vegetable crops, including citrus, tomatoes, onions, carrots and lettuce [2]. It is also present in raw milk and has long been associated with dairy spoilage. Since the 1980s, however, *G. candidum* has been utilized by the commercial cheesemaking industry as a ripening culture that influences flavour and determines the appearance of the rind [1]. It promotes the growth of *Pencillium camemberti* via the metabolism of lactate and may inhibit the growth of undesirable organisms such as *Mucor* spp. and *Listeria monocytogenes *.

Like *Candida*, *G. candidum* is a commensal organism known to colonize the human skin, tracheobronchial tree and gastrointestinal tract [3–5]. It is a rare human pathogen that is considered to have low virulence but can cause local or disseminated disease referred to as geotrichosis [3, 6–8]. The organism is usually acquired via ingestion or inhalation [9]. The primary risk factor for geotrichosis is being immunocompromised, namely neutropenia, secondary to haematological malignancy or cytotoxic chemotherapy [10]. HIV [11], corticosteroids [12], diabetes mellitus [13], altered gut mucosal immunity [5], indwelling catheters [8, 14], parenteral nutrition, prolonged use of broad-spectrum antibiotics [3], alcohol abuse, chronic lung or kidney disease and critical illness [15] have also been implicated as risk factors.

The presentation of geotrichosis is highly variable due to underlying host factors. Disseminated cases, usually with fungaemia [3, 7–10, 12], and localized disease involving the skin [13], brain [16], eye [17, 18], lacrimal sac [19], oral mucosa [4, 11], esophagus [20], stomach and duodenum [5], ileum [6], lung [12, 21], and kidney [22] have been reported in the literature. Trauma may be the only predisposing factor for the development of geotrichosis in an immunocompetent, otherwise healthy patient. Hrdy *et al*. described a case of metacarpophalangeal (MCP) joint infection due to *G. candidum* following traumatic inoculation by a splinter in a 27-year-old man with no medical problems [23]. Invasive lung infection due to other *Geotrichum* spp. has also been described after trauma [24]. This genus remains an uncommon but under-recognized cause of opportunistic infection in burn patients [25]. Here we report the first case, to our knowledge, of cutaneous geotrichosis due to *Geotrichum candidum* in a patient with severe thermal burns.

## Case

A patient in their mid-20s with cognitive impairment due to cerebral palsy presented to the emergency department (ED) as a trauma code with approximately 30 % total body surface area (TBSA) flame burns sustained while reaching over a gas stove, which set their shirt alight. The patient’s grandmother extinguished the fire with water and applied peroxide, and then brought them to the hospital where they were admitted to the intensive care unit (ICU) under the Burn Surgery service.

At presentation, the patient was afebrile, with a blood pressure of 124/86 mmHg, a heart rate of 151 beats min^−1^ and SpO_2_ of 96 % on room air. A physical examination revealed 28 % TBSA third-degree burns involving the right external ear, neck, anterior and lateral trunk and bilateral upper arms. There was also a 2 % TBSA second-degree burn to the left anterior thigh. The remainder of the examination was unremarkable. The patient was found to have acute renal failure with a creatinine of 1.67 mg dl^−1^ (0.4–1.1 mg dl^−1^), as well as elevated lactic acid of 3.6 mMol l^−1^ (0.4–2.0 mMol l^−1^) and a total leukocyte count of 34.2×10^9^ cells l^−1^ (3.5–10.6×10^9^ cells l^−1^). Blood cultures and urinalysis were negative.

The patient was started on broad-spectrum antimicrobial therapy with vancomycin, cefepime and fluconazole for sepsis in the setting of their extensive severe burns. They were taken to the operating room (OR) shortly after admission for excisional debridement with autografting and xenografting of the wounds. On hospital day 9, the patient underwent repeat excisional debridement and autografting from the buttocks and lower extremities to the face. Tissue cultures from the face grew methicillin-resistant *Staphylococcus aureus* (MRSA), *Candida orthopsilosis* and *Acinetobacter baumannii* resistant only to gentamicin and ertapenem.

The patient was intubated on hospital day 12 due to hypotension, bradycardia, hypothermia (Tmin 34.2 °C) and desaturation. They were diagnosed with multifocal *A. baumannii* pneumonia and treated with ampicillin/sulbactam. On hospital day 14, blood culture (BD BACTEC FX) grew *C. orthopsilosis*, which was thought to be line-related and treated with micafungin. A funduscopic examination was negative for candida retinitis.

On hospital day 15, the patient underwent further debridement and autografting due to the failure of the prior graft material. Tissue cultures from the right flank grew two yeasts: *C. orthopsilosis* (blood agar) and, later, *Geotrichum candidum* (Sabouraud dextrose agar). They were identified by matrix-assisted laser desorption/ionization time-of-flight mass spectrometry (MALDI-ToF MS). Susceptibility results for the initial blood isolate of *C. orthopsilosis* performed by Etest (bioMérieux, Marcy l' Étoile, France) revealed that it was susceptible to voriconazole (MIC 0.5 μg ml^−1^), micafungin and amphotericin B (MIC 0.25 μg ml^−1^ for both drugs). Send-out MIC testing by Etest was also requested for the *G. candidum* isolate (ARUP Labs, Salt Lake City, UT, USA). The patient was successfully extubated on hospital day 16. Their right internal jugular (IJ) central venous catheter (CVC) was exchanged over a guidewire with negative subsequent blood cultures.

On hospital day 18, the patient developed bilious emesis, respiratory distress and fever. They were reintubated and diagnosed with pneumonia due to MRSA and *Escherichia coli* resistant to ampicillin/sulbactam and cefepime. Ampicillin/sulbactam was therefore changed to meropenem. Due to the severity of the patient’s illness and pending MIC data for *G. candidum*, micafungin was also changed to voriconazole.

**Fig. 1.**
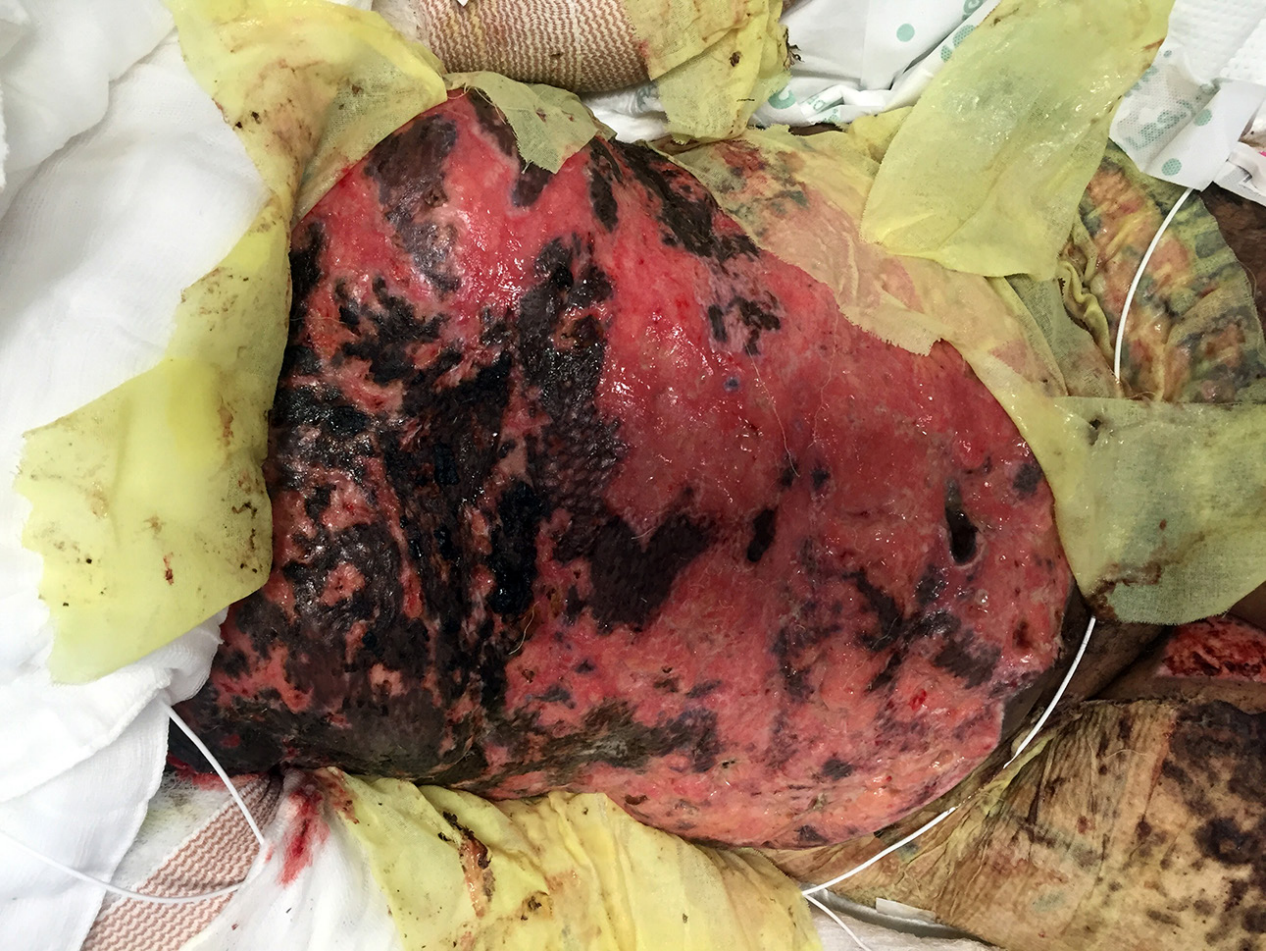
Failed anterior torso skin graft on hospital day 28.

By hospital day 26, the majority of the patient’s allografts had failed (Fig. 1). They developed several episodes of fever (Tmax 38.3 °C) with persistent leukocytosis (18.3×10^9^ cells l^−1^). Due to the patient’s worsening clinical condition, voriconazole was switched to liposomal amphotericin B for better empirical coverage of the previously isolated *G. candidum*. On hospital day 30, the antifungal MICs of this organism resulted (see Table 1). Due to the high amphotericin B MIC and several episodes of emesis, this agent was switched back to a combination of voriconazole and micafungin (for *C. orthopsilosis*).

**Table 1.** Antifungal MICs of the patient’s *G. candidum* isolate

**Antifungal medication**	**MIC (μg ml^−1^)**
Fluconazole	32
Itraconazole	1.0
Posaconazole	1.0
Voriconazole	0.5
Isavuconazole	0.125
Amphotericin B	2.0
Flucytosine	0.25
Micafungin	0.5
Caspofungin	1.0
Anidulafungin	2.0

The patient again became febrile on hospital day 33 and was found to have line-related MRSA bacteraemia, which was treated with vancomycin. Following further debridement and autografting on hospital day 36, most of the prior grafts were noted to have healed sufficiently for the left IJ CVC to be replaced with a right brachial midline.

The hospital course was further complicated by catheter-associated cystitis due to *Proteus mirabilis* and *Achromobacter xylosoxidans *, which were treated with 5 days of trimethoprim/sulfamethoxazole. A percutaneous endoscopic gastrostomy (PEG) tube was placed for malnutrition. The patient received a total of 6 weeks of antifungal therapy while an inpatient, with eventual excellent healing of their burn wounds. They were discharged to a rehabilitation facility on hospital day 64 and made a full recovery.

## Discussion

The above case highlights the complexity of recognizing and managing infections, particularly invasive fungal infections, in burn patients. These individuals can be considered to be immunocompromised by virtue of their extensively disrupted integument and impaired cell-mediated immunity [26]. Measures designed to protect the skin and promote wound healing, including external heat sources and moisturized dressings, may inadvertently promote fungal growth [27]. Prophylactic antibiotics are not recommended in burn patients due to their lack of efficacy in preventing colonization, infection or toxic shock, and their propensity to cause overgrowth of other bacteria [26, 28]. Broad-spectrum antibiotics used for other indications may also facilitate the overgrowth of typically non-pathogenic fungi by altering the normal host microbiome [3]. Commensal organisms such as *Geotrichum* spp. can then enter the body through the thermally injured skin to cause soft tissue infection or more severe invasive disease. Pathogens can also be introduced nosocomially or by pre-hospital water exposure [27]. TBSA burns ≥40 %, inhalational injury, the presence of CVCs and advanced age have all been associated with increased risk of fungal infection in burn patients.

This patient experienced multiple infections with drug-resistant pathogens throughout their hospitalization. Many of their skin grafts failed despite treatment with broad-spectrum antibiotics, raising the concern that the untreated *G. candidum* isolate could be a true pathogen. The organism had already been identified in this case, but in general the greatest challenge in cases of suspected invasive fungal infection is prompt and accurate diagnosis of the aetiological agent. Histopathological examination or microscopic examination of a culturable fungus is essential and may provide identification based on morphology alone [4]. However, the hyphae of young *G. candidum* cultures may be indistinguishable from other fungi, including *Candida*, *Trichosporon* and *Aspergillus* spp. [6, 7]. In such cases further *in* *vitro* biochemical or molecular testing is required to make the diagnosis. Table 2 provides a summary of these organisms with regard to morphology, biochemical characteristics, useful laboratory testing and treatment.

**Table 2.** Comparison of the morphology, biochemical characteristics, diagnostic testing and treatment of *G. candidum*, *Candida*, *Trichosporon* and *Aspergillus* [2, 5–7, 14, 17, 29–34]

		***Geotrichum candidum***	***Candida* spp**.	***Trichosporon* spp**.	***Aspergillus* spp**.
Morphology		Hyaline, septate true hyphae with irregular branching, which segment to form arthroconidia of variable lengths (4–10 µm)	Blastoconidia, pseudohyphae or septate true hyphae	Septate true hyphae, pseudohyphae, arthroconidia and blastoconidia	Uniform septate hyphae, dichotomous acute angle branching (45°), terminal conidiophores
Arthroconidia		+	−	+	Conidiophores
Blastoconidia		−	+	+	
Staining	Haematoxylin/eosin	Stains poorly	Stains well	Stains well	Stains well
	Methenamine silver	Deep staining of hyphal wall, empty-appearing core	Uniform hyphal staining	Uniform hyphal staining	Uniform hyphal staining
Colony appearance (Sabouraud agar)		White to cream, hairy, rapid growth	White to cream, smooth, rapid growth	White to cream, farinose, often with cerebriform features or radial furrows	Velvety, powdery or woolly. Pigment varies by species. *A. fumigatus*, white to blue-green; *A. niger*, black; *A. flavus*, yellow to green; *A. terreus*, brown
Carbohydrate assimilation		Glucose, galactose, xylose. Not lactose, maltose or sucrose	Variable. All assimilate glucose. Most assimilate maltose, sucrose, galactose and trehalose	Glucose, galactose, sucrose, maltose, lactose	Glucose, sucrose, maltose, fructose, lactose (not *A. niger*)
Urease		−	−	+	+
Diagnostic testing		Insufficient evidence; see text	*β*-d-glucan T2Candida panel	Dual *Cryptococcus* antigen and galactomannan positivity is suggestive, sensitivity unknown	Galactomannan, PCR
Treatment		Amphotericin B±flucytosine or voriconazole alone	Echinocandin or triazole	Superficial: shaving+topical triazole or selenium sulfide Invasive: voriconazole±amphotericin B	Amphotericin B, voriconazole or isavuconazole

**Fig. 2:**
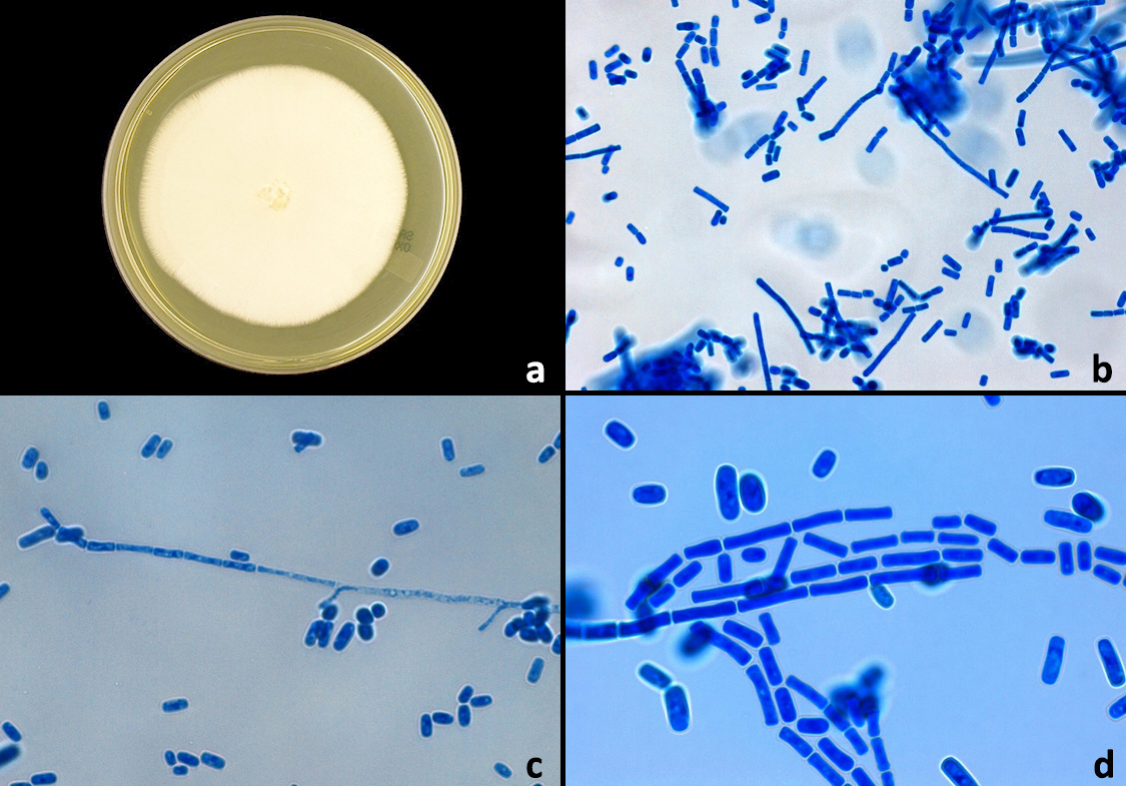
(a) *G. candidum* grown on Sabouraud agar at 30 °C for 5 days. (b) Lactophenol cotton blue (LPCB) wet mount of *G. candidum* at 400× magnification. (c) Hyaline hypha with developing arthroconidia (left) and separate individual arthroconidia (400×). (d) Chains of arthroconidia and separate arthroconidia (1000×+10 % digital magnification). Images courtesy of Yuri Amatnieks.

The genus *Geotrichum*, the taxonomy of which was defined in 2004 by de Hoog and Smith, is composed of 22 species [33, 35]. The best-known of these is *G. candidum*, a yeast which on Sabouraud agar appears as rapidly growing, white to cream, hairy colonies (Fig. 2a) [17, 33]. It grows optimally at 25°C. On chromogenic culture media (e.g. Chromogenic Candida Agar; Oxoid, Basingstoke, UK) the organism can be differentiated from *Candida* spp. based on its distinct pink colour and colony morphology [4, 36]. Microscopically, *G.* *candidum* forms hyaline, septate true hyphae with irregular branching that segment to form arthroconidia of variable lengths (4–10 μm) (Fig. 2b–d) [2, 12, 17]. Blastoconidia are absent. It assimilates glucose, galactose and xylose, but not lactose, maltose or sucrose [13].

Two other *Geotrichum* spp. are recognized as human pathogens: *Geotrichum capitatum* and *Geotrichum clavatum*. The former can be differentiated from *G. candidum* by the formation of annelloconidia, the ability to grow at 45 °C and resistance to cycloheximide [37]. While galactomannan and (1-3)-*β*-d-glucan positivity has been reported in the setting of *G. capitatum* infection, there is no literature to support the use of these tests to detect *G. candidum* [38, 39]. MALDI-TOF MS or M13 microsatellite PCR can be used to differentiate between *Geotrichum* spp., while sequencing of 18S rDNA or the 5.8S region and its adjacent internal transcribed spacers (ITS1-5.8S-ITS2) has been used to characterize different strains within the same species [1, 14, 40].

Before *G. candidum* was recognized as a potentially invasive pathogen, experts debated the need for treatment [12]. One case of *G. candidum* fungaemia responded well to the removal of a central venous catheter in the absence of antifungal therapy; the percentage of circulating neutrophils containing arthroconidia decreased from 48 to 0 % following removal of the device [3]. By the mid-1970s, however, it became evident that this organism could disseminate and cause significant morbidity and mortality. Multiple therapies met with some success, including potassium iodide, colistin, neomycin sulfate, tetracycline and nystatin [3, 12]. Prolonged oral nystatin has been used to effectively manage superficial oral geotrichosis in the setting of HIV [11]. Amphotericin B had become the treatment of choice for invasive disease by the late 1980s, with or without adjunctive antifungal medications [8].

Due to antifungal MIC variability among *G. candidum* isolates and among other fungal pathogens with which it may be confused, *in vitro* MIC determination is essential to guide effective treatment [6]. Results must be interpreted with caution since MICs may vary based on methodology, and there are no current established antifungal breakpoints for *Geotrichum* spp. Voriconazole has consistently been found to have the lowest *G. candidum* MICs among the azoles [8, 13, 17]. The organism generally has low amphotericin B, itraconazole, posaconazole and flucytosine MICs, while it demonstrates elevated MICs to fluconazole [13, 15]. *G. candidum* was reported to be ‘resistant’ to flucytosine in one study [7]. The echinocandins have variable reported activity against *Geotrichum* spp., with MICs ranging from 0.06 μg ml^−1^ to >8 μg ml^−1^ [8, 15, 41, 42]. Despite possible *in vitro* activity, these agents are not recommended for the treatment of geotrichosis.

Amphotericin B MICs to *G. candidum* have also varied in the literature, with several studies reporting MICs of as high as 2 μg ml^−1^ [15, 42]. When liposomal amphotericin B was tested directly against the organism, however, the MICs ranged from 0.06 to 0.44 μg ml^−1^, suggesting that tissue concentrations of the newer formulations of this drug would be sufficient to overcome relative *in vitro* ‘resistance’ [42]. That said, extremely high minimum fungicidal concentrations (MFCs) have been observed for all tested antifungals to *G. candidum*, highlighting the importance of host defences in controlling infections due to this organism and other fungi. The optimal antifungal treatment of geotrichosis has not been assessed in clinical trials [14]. Based on the available data, the recommended first-line treatments include amphotericin B with or without flucytosine, or voriconazole alone [14, 15]. Sequential treatment with amphotericin B followed by an oral triazole has been used successfully [13]. Newer azoles, including posaconazole and isavuconazole, are promising potential therapies, although breakthrough infection has been reported with posaconazole [15].

Our patient’s case was particularly challenging due to the presence of multiple fungal pathogens with different susceptibility profiles. They experienced both cutaneous *G. candidum* infection involving their burn wounds and *C. orthopsilosis* fungaemia related to a central line. Amphotericin B was used to treat both until an elevated *G. candidum* MIC for this agent was identified. The patient was also unable to tolerate a prolonged amphotericin course due to GI side effects and was therefore stepped down to voriconazole plus micafungin. Voriconazole could have been used alone, but the echinocandin was continued due to potential additive or synergistic activity against *G. candidum* as well as for treatment of the patient’s candidaemia. Despite the low micafungin MIC, this agent may not have been active at all, since the patient worsened clinically during therapy and *G. candidum* was also isolated after its initiation. Isavuconazole is another theoretical monotherapy, but did not meet the criteria for noninferiority to echinocandins for the treatment of candidemia, and is thus not approved for this indication [43].

### Conclusion

Here we describe the first known case of cutaneous *G. candidum* infection in a patient with severe thermal burns. The main risk factor for invasive fungal infection, including geotrichosis, is neutropenia due to haematological malignancy or chemotherapy [10]. Other forms of immunocompromise (e.g. HIV, steroid/immunosuppressant use, diabetes mellitus, alcoholism, critical illness) also predispose to geotrichosis. In burn patients, disruption of the integumentary barrier allows for invasion by commensal fungi. Prolonged broad-spectrum antibiotic use may promote fungal overgrowth [3].

Aggressive source control through tissue debridement or removal of infected indwelling catheters, as occurred in this patient, is essential in the management of geotrichosis [3, 23]. Since host defences are also crucial in controlling infection, reducing immunosuppression may be helpful [42]. Concerted efforts should be made to identify the causative organism promptly and subsequent MIC determination should be performed to guide antifungal therapy. The current recommended treatments for geotrichosis include amphotericin B with or without flucytosine or voriconazole alone [14]. Echinocandins should be avoided.

*Geotrichum* spp. are rare emerging fungi which, despite adequate antifungal therapy, are associated with a mortality rate approaching 50 % [15]. Clinicians should be aware of geotrichosis as a clinical entity in burn patients as well as the immunocompromised. Antifungal resistance and breakthrough disease are an ongoing concern due to the increasing number of at-risk patients and the use of routine mould prophylaxis [14, 44].
